# Effect of caffeinated gum on a battery of rugby-specific tests in trained university-standard male rugby union players

**DOI:** 10.1186/s12970-019-0286-7

**Published:** 2019-04-11

**Authors:** M. K. Ranchordas, H. Pratt, M. Parsons, A. Parry, C. Boyd, A. Lynn

**Affiliations:** 1 Academy of Sport and Physical Acitivty, Faculty of Health & Wellebing, Sheffield Hallam University, Sheffield, S10 2BP UK; 2 Food Group, Sheffield Business School, Sheffield Hallam University, Sheffield, UK

**Keywords:** Caffeine, Gum, Rugby, Jump, Sprint, Running, Endurance, Yo-Yo, Fatigue

## Abstract

**Background:**

Caffeine has been shown to enhance strength, power and endurance, characteristics that underpin performance in rugby. Caffeinated gum has attracted interest as a novel vehicle for delivering caffeine, because absorption of caffeine from gum is quick. Rapid absorption of caffeine may be useful during rugby matches when there is limited time for supplementation such as at half-time or when substitutes enter play. The purpose of this study was to determine whether a low dose of caffeine in gum improves performance in a battery of rugby-specific tests.

**Methods:**

In a double-blind, randomized, placebo-controlled, crossover design, 17 male university-standard rugby players (mass: 85.6 ± 6.3 kg; height: 179.4 ± 6.2 cm; age: 20.4 ± 1.2 years) chewed caffeinated gum (200 mg caffeine) or a placebo gum on two occasions separated by a week. After a standardized warm-up, gum was chewed for 5 min. Subsequently, participants performed three countermovement jumps, followed by an Illinois agility test, 6 × 30 m repeated sprints, and the Yo-Yo IR-2 test; each test was separated by short rest periods.

**Results:**

Caffeinated gum enhanced countermovement jump by 3.6% (caffeine: 43.7 ± 7.6 cm vs. placebo: 42.2 ± 6.2 cm; *d* = 0.22, 95% CI [0.006, 0.432]; *p* = 0.044). There was a greater resistance to fatigue during the 6 × 30 m repeated sprint test (fatigue index caffeine: 102.2 ± 0.9% vs. placebo: 103.3 ± 1.2%; *d* = 1.03, 95% CI [0.430, 1.613]*; p =* 0.001), and performance on the Yo-Yo IR2 was improved by 14.5% (caffeine: 426 ± 105 m, placebo: 372 ± 91 m; d = 0.55, 95% CI [0.130, 0.957]; *p* = 0.010). Caffeine gum had no significant effect on the Illinois agility test (caffeine 16.22 ± 1.08 s vs. placebo 15.88 ± 1.09 s; *d* = − 0.31, 95% CI [− 0.855, 0.240]; *p* = 0.271).

**Conclusions:**

In university-standard rugby players, a low dose of caffeine (200 mg) supplied in chewing gum enhanced performance on the Yo-Yo IR-2 test and the countermovement jump test and reduced fatigue index during repeated sprints. These improvements in a battery of rugby-specific tests may transfer to enhanced performance in rugby matches.

## Background

Caffeine is one of the most commonly consumed ergogenic aids as evidenced by a study of elite athletes, which detected caffeine in 74% of urine samples collected at doping controls [1]. The popularity of caffeine among athletes is understandable, because it has been shown to enhance: endurance performance [2], high intensity activity (1–60 min duration) [3], and performance during stop-and-go activities [4, 5] that typify team sports [6]. Caffeine is believed to exert its ergogenic effects largely as a result of blocking adenosine receptors in the central nervous system [7]. Evidence indicates that this enhances performance through increasing central drive and alertness and reducing muscle pain and rating of perceived exertion [7, 8].

Caffeine has multiple performance enhancing effects. It has been shown to reduce reaction time [9] and increase: anaerobic power [5], sprint speed [10] and endurance. Rugby is a sport that may benefit from the multiple ergogenic effects of caffeine. A rugby match is characterised by high-intensity running interspersed with bouts of low activity, multiple changes of direction and technical skills [11]. Strength and power are also key requirements especially when players are engaged in tackles, scrums, rucks and mauls [12]. Rugby players cover between 4000 and 7000 m during a typical match, so considerable endurance is also needed to ensure performance is maintained during game defining moments [13–15].

Previous studies have investigated the effects of caffeine on rugby-related performance measures such as sprints and impacts [16], and on rugby specific test protocols [17]. Del Coso et al. reported beneficial effects of a caffeine containing commercial energy drink (3 mg·kg^− 1^ of body weight) on running pace and pace at sprint velocity during 3 games of rugby sevens and on power output in a 15 s jump test performed after the last game. Similarly, Stuart et al. found that 6 mg·kg^− 1^ of caffeine (in a capsule) improved performance across a range of rugby specific tests (sprint speed, first and second drive power and passing accuracy) during a 2 × 40 min simulated rugby performance test.

Traditionally, caffeine has been provided in a capsule or beverage approximately one hour prior to exercise [3, 18–20]. However, chewing gum offers a novel alternative vehicle for the delivery of caffeine. The absorption of caffeine from chewing gum is more rapid than from capsules (5–10 min versus 45–60 min) [21]. This is because caffeine absorption from chewing gum initially occurs through the buccal mucosa [22], an accepted route for accelerating drug absorption [23]. Because drug action is limited by absorption rate, caffeine gum would be expected to have a faster onset of action than capsules [22]. The use of caffeine gum may therefore have practical applications to rugby, where limited time for nutritional interventions exist (e.g., the end of the warm-up, at half-time, or for substitutes required to enter competition with limited notice).

Despite the potential practical benefits of caffeine gum, few studies have investigated its effect on team sports. In a study of professional academy rugby players, 400 mg of caffeine delivered in gum failed to rescue the decrement in performance across two sprint tests (6 × 40 m) separated by a 15 min simulated half time period [24]. This lack of effect may have been because the rugby players had a mean habitual intake of caffeine of 191 ± 138 mg·d^− 1^ [25, 26]. Evans et al. reported that caffeine gum reduced the decrement in repeated sprint performance in team sports players with a low caffeine intake but not in those with an intake greater than 130 mg·d^− 1^. In contrast, despite supplying a lower dose of caffeine (200 mg) Ranchordas et al. [4] found small, but statistically significant positive effects of caffeine gum on two soccer specific performance tests (Yo-Yo Intermittent Recovery Test Level 1 and countermovement jump) in university-standard soccer players. The disagreement between studies may be explained by differences in study design, performance tests and characteristics of the participants. Given the possible practical benefits of delivering caffeine rapidly during competitions, further studies are needed to clarify the effects of caffeine gum on team sport specific performance tests. Therefore, this study was conducted to determine the effect of caffeine gum on a battery of rugby-specific performance tests. The effect of a low 200 mg dose of caffeine was investigated because: (i) it had previously been shown to be adequate to improve performance in soccer players [4]; (ii) to minimize the risk of adverse effects and (iii) to be consistent with the manufacturer’s recommendation for use.

## Methods

### Design

A randomised, double-blind, placebo-controlled, crossover design was used to examine the effectiveness of caffeine gum on a battery of rugby-specific tests. Participants were randomly allocated and counterbalanced into caffeine and placebo groups using a random sequence generator (Graphpad Software Inc. California, USA). Data was collected between February and March 2017 during the University’s competitive rugby season.

### Participants

The sample size was estimated using the data from a previous study that investigated the effects of caffeine on jumping and sprint performance in rugby players. The α-level was set at 0.05 and power (1 − β) at 0.8 for differences in running performance between conditions and calculated that a sample size of 20 was necessary. Twenty male competitive university-standard rugby players were recruited, but three participants dropped out before the start of the study; one due to personal reasons and two because of injury. Thus, seventeen participants completed this study (mean ± SD; mass: 85.6 ± 6.3 kg; height: 179.4 ± 6.2 cm; age: 20.4 ± 1.2 years) with a minimum of three years’ experience of playing competitive rugby. Inclusion criteria required weekly training of approximately 3–4 h and one competitive match lasting 80 min every week or every other week. At the time of the study all participants were free from illness and injury. Volunteers that were sensitive to caffeine (assessed via a caffeine sensitivity questionnaire) or suffering from any major or minor injuries or medical complications were excluded via a medical pre-screening questionnaire. Ethics approval was obtained from Sheffield Hallam University’s Sport and Physical Activity’s Ethics Committee and participants completed a pre-screening questionnaire and provided written informed consent before commencing the study.

### Procedures

Participants were instructed to replicate their diet and physical activity 48 h before each testing session and were given a diary to record this information. In addition, participants were asked to avoid alcohol, strenuous exercise and consume no more than 100 mg of caffeine per day for 48 h before each trial. Food and activity records were checked for compliance and the researchers were satisfied that the instructions were adhered to. Due to the training and match schedule of the rugby club, player availability was agreed in advance with the head coach to allow for a familiarization trial and two further experimental sessions across a 14-day period. There were 7 days between each experimental session to allow sufficient time for recovery. Participants attended three sessions at the Sheffield Hallam University indoor sports hall. The first session was a familiarization session with no supplements to habituate participants to the testing protocols. For the remaining two sessions, participants completed the same testing protocols with supplementation of either caffeine gum or placebo gum. Testing sessions took place at the same time of day to control for diurnal variation. Immediately prior to the familiarization trial, body mass (kg; HD-327 digital scales, Tanita, Japan), stature (m; Leicester height measure; Invicta Plastics Limited, UK) and playing position were recorded and participants completed a Physical Activity Readiness Questionnaire. Participants were asked to wear the same clothing and footwear to all the testing sessions.

### Caffeine gum and placebo

The experimental gum was mint flavoured, sugar free (sweetened with sorbitol) and contained 100 mg of caffeine per piece (Superfast Energy Gum™^;^ Rotherham, UK). The placebo gum was provided by the manufacturer and was matched for taste and appearance but was free of caffeine. Participants were instructed to chew two pieces of gum (200 mg of caffeine which was equivalent to 2.3 ± 0.2 mg·kg^− 1^ of bodyweight) for 5 min immediately after the warm-up. It has been previously reported that 85% of the dose is released within this time-frame [21]. Chewing was timed by the researchers using a handheld stopwatch.

### Rugby-specific tests

**Fig. 1:**
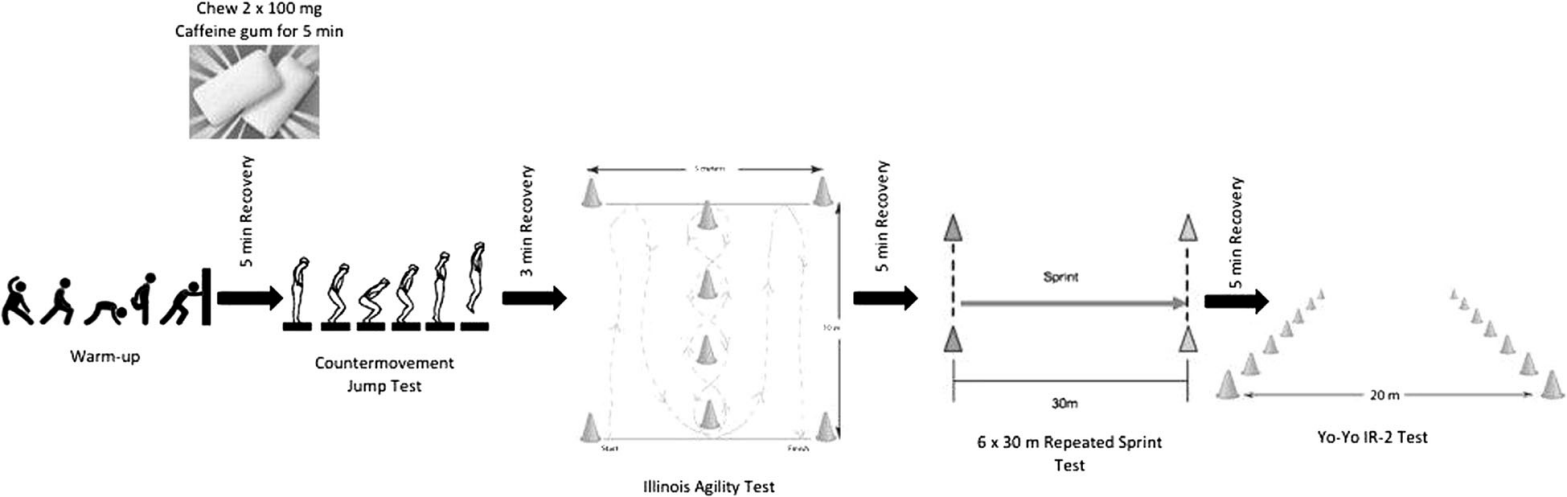
A schematic diagram of the order of the testing battery and the timing of the supplement

A fingertip blood sample was collected from each participant on arrival. Blood lactate was determined on an EKF Diagnostic Biosen C/line (UK) autoanalyser (within-day coefficient variance of < 1.5%, at a value of 12 mmol/L). Participants completed a standardized 10 min rugby-specific warm-up led by their conditioning coach. This consisted of jogging, 10 m sprinting, speed/agility drills and dynamic stretches. This was followed by three 20 m runs at instructed intensities of effort of 50, 70 and 90%. Figure 1 provides a timeline for order and the rest periods of the testing protocols.

Following the warm-up, participants carried out the countermovement jump test (CMJ) using an optical measurement system (Optojump Next; Microgate; USA). The CMJ is a triple extension movement which is specific to rugby and it is used to measure an athlete’s jump height which has been identified as a valid and reliable indicator of power [27]. The CMJ required the participants to stand upright then drop down into the squat position and then immediately jump as high as possible. The participants were given one practice jump followed by three maximal jumps separated by 1 min recovery between jumps and the highest jump height was recorded.

Three minutes after the CMJ test, players moved on to the Illinois agility test. This field test has been reported to be a reliable and valid test of an athlete’s ability to change direction quickly [28]. The test was completed in an area of 10 × 5 m and the participants followed the course depicted in Fig. 1. Participants had one practice and then completed the circuit once as quickly as possible. The time from start to finish was recorded using timing gates (Brower timing systems; Draper, Utah, USA).

After a five min rest, participants completed the 6 × 30 m repeated sprint test. Timing gates (Brower timing systems; Draper, Utah, USA) and cones were placed at the 0 and 30 m points to record the sprint times. After each sprint, participants jogged back to the start within 20 s (Fig. 1); this procedure was then repeated 6 times. This test is a modified version of the repeated sprint test put forward by Spencer et al. [29] which has been shown to have high levels of validity and reliability, but, here, has been modified to suit the needs of a rugby game.

After a final 5 min recovery period, the Yo-Yo IR2 test [30] was performed. This test required the participants to carry out 2 × 20 m shuttle runs that gradually increased in speed as dictated by audio signals. Each run was separated by 10 s active recovery where participants jogged around a cone positioned 5 m behind the start line. This active recovery period is congruent to the endurance demands of a rugby match. Two consecutive failures to reach the finish line before the audio signal indicated test cessation and the distance covered at that point was the final test result. All tests were performed with verbal encouragement from the coaching staff which was replicated for all trials to ensure consistency. Average and peak heart rates were monitored throughout the Yo-Yo test protocol (Activio Sport System, version 3.1.0.21). Immediately after the Yo-Yo IR2 test, the level achieved was recorded and each individual’s rating of perceived exertion (RPE) for the test was assessed using a Borg scale [31], and blood lactate concentration was measured.

### Statistical analysis

Statistical analyses were performed using SPSS 24.0 for Windows (IBM, Chicago, IL) and Exploratory Software for Confidence Intervals (ESCI, https://thenewstatistics.com/itns/esci/). Repeated measures ANOVA was used to assess treatment effects on the 6 × 30 m repeated sprint test. Treatment effects on all other outcome measures were assessed using paired sample *t* tests. Paired data and standardised residuals were normally distributed as assessed by the Shapiro-Wilks test. Standardised effect sizes were calculated using SPSS for ηp^2^ and ESCI for Cohen’s *d*. Effect sizes were interpreted using the classifications of 0.2, 0.5 and 0.8 as small, moderate and large effects, respectively [32]. Statistical significance was set at *p* ≤ 0.05 and data are expressed as mean values ± SDs. Mean differences and Cohen’s *d* values are displayed with 95% CI.

## Results

### Side effects and effectiveness of blinding

At the end of the final testing session participants were asked to guess the order of supplementation, but only four participants correctly identified the order, suggesting that blinding was effective. No adverse responses to the caffeine gum or placebo were reported.

### Countermovement jump and Illinois agility performance

**Fig. 2:**
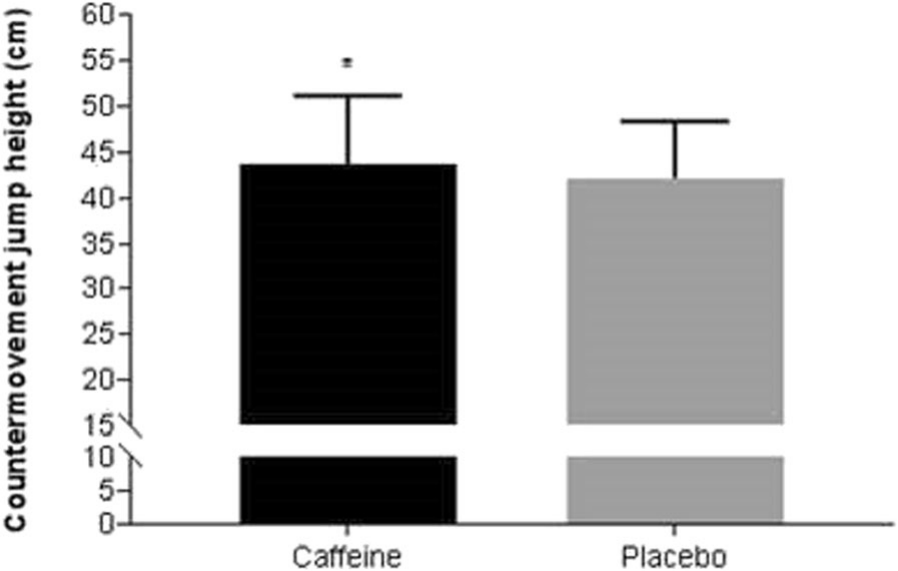
Countermovement jump height (*n* = 17). Data are expressed in mean ± SD. * Caffeine significantly higher than placebo (*p* = 0.044)

Caffeine gum evoked a significant improvement in countermovement jump height of 1.5 cm (95% [CI 0.05, 3.00]; caffeine: 43.7 ± 7.6 cm, placebo: 42.2 ± 6.2; *p* = 0.044) (Fig. 2). Cohen’s *d* revealed a small effect (*d* = 0.22; 95% CI [0.006, 0.432]).

No statistically significant differences were observed for the Illinois agility test. Results for the two conditions were 15.88 ± 1.09 s for placebo and 16.22 ± 1.08 s for caffeine (mean difference 0.34 s, 95% CI [− 0.29, 0.97]; *p* = 0.271). Cohen’s *d* revealed a small effect size in favour of placebo (*d* = − 0.31, 95% CI [− 0.855, 0.240]).

### Repeated sprint performance

**Fig. 3:**
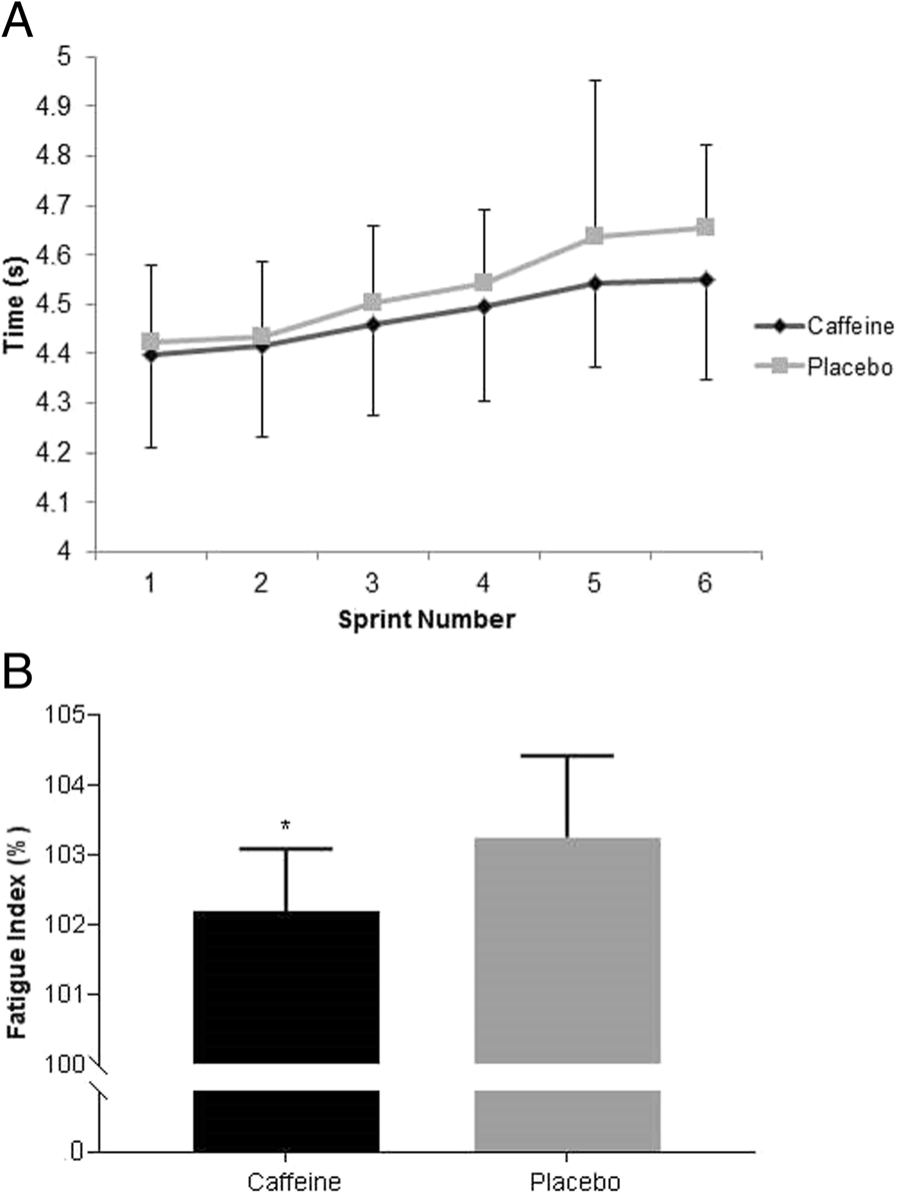
(**a**) 6 × 30 m sprint times (n = 17). Data are expressed in mean ± SD. **b** Fatigue Index (n = 17). Data are expressed in mean ± SD. * Caffeine significantly lower than placebo (*p* = 0.001)

Participants exhibited a statistically significant decline in sprint performance over the six repeated sprints (main effect for time, *p* < 0.001; *ηp*^2^ = 0.476). There was no statistically significant difference in sprint performance between the two conditions (*p* = 0.341; *ηp*^2^ = 0.028), and there was no statistically significant condition*time interaction (*p* = 0.256; *ηp*^2^ = 0.042; Fig. 3a).

Calculation of a Fatigue Index (FI = ([sum of all sprints / fastest sprint × 6] × 100) -100) revealed a higher FI for the placebo in comparison to the caffeine condition (caffeine: 102.2 ± 0.9%, placebo: 103.3 ± 1.2%; mean difference − 1.07 95% CI [− 1.60, − 0.54], *p* = 0.001; Fig. 3b). The caffeine gum condition resulted in a greater resistance to fatigue, permitting a more sustained capacity for repeated sprint performance (large effect, *d* = 1.03, 95% CI [0.430, 1.613]).

### Yo-Yo-IR2 test, blood lactate and heart rate

**Fig. 4:**
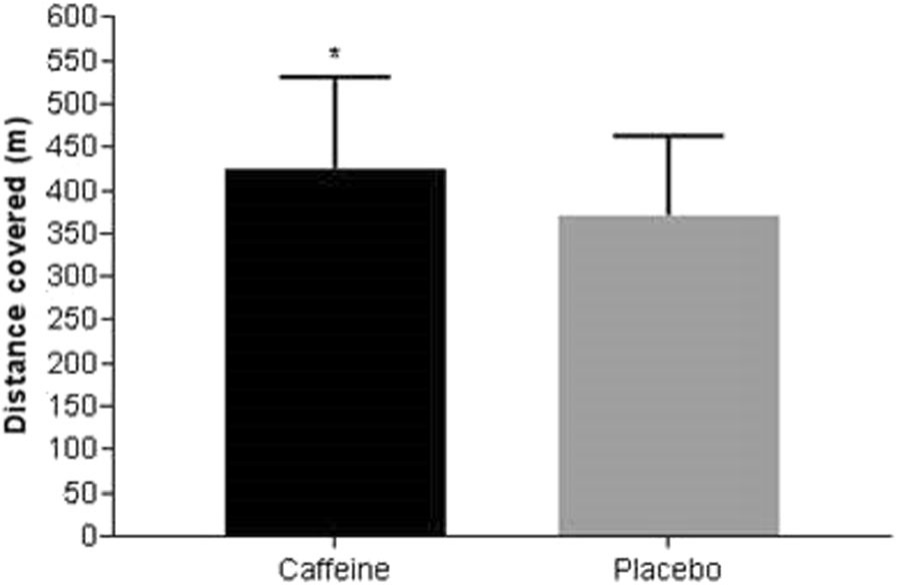
Total distance covered on the Yo-Yo intermittent recovery test level 2 (n = 17). Data are expressed in as mean ± SD. * Caffeine significantly higher than placebo (*p* = 0.010)

**Fig. 5:**
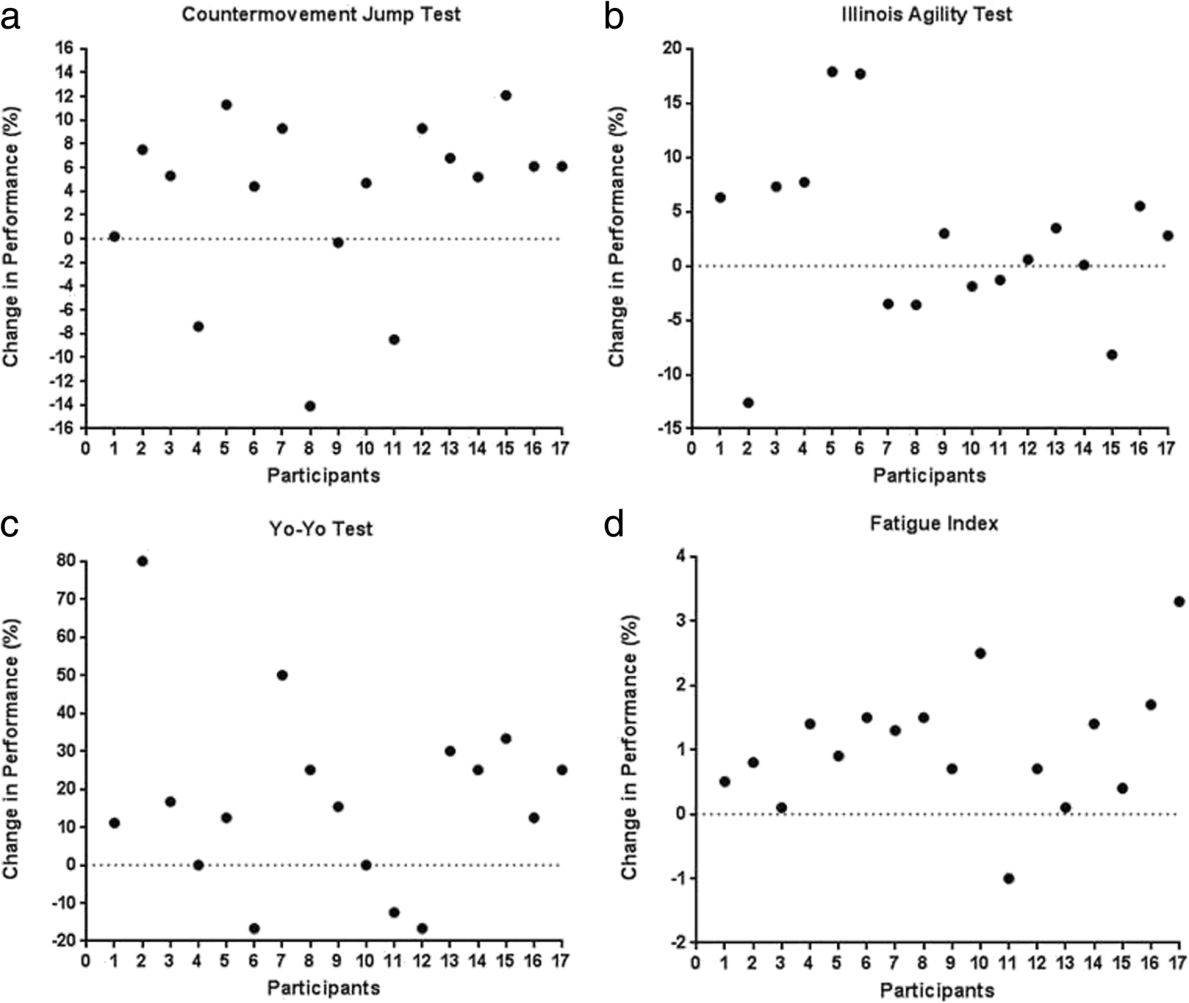
Individual responses to caffeine gum supplementation on the battery of tests (n = 17). expressed in percentage change from baseline. **a** - Countermovement Jump Test; **b** - Illinois Agility Test; **c** - Yo-Yo Test; **d** - Fatigue Index

The results of the Yo-Yo test (Fig. 4) revealed that participants covered 54.1 m (95% [CI 15.00, 93.23](caffeine: 426 ± 105 m, placebo: 372 ± 91 m; *p* = 0.010) more distance after ingestion of the caffeine gum than the placebo gum (medium effect, *d* = 0.55, 95% CI [0.130, 0.957]; *p* = 0.010). The improvement in performance of one player (participant number 2 see Fig. 5) after caffeine was much greater (80%) than other participants. Removal of this participant’s data slightly attenuated the ES from 0.55, 95% CI [0.130, 0.957] to 0.50, 95% CI [0.075, 0.906] but the effect of caffeine remained statistically significant (*p* = 0.020).

Although blood lactate was higher in the caffeine condition at the end of the test, the difference was not statistically significant (caffeine: 14.4 ± 3.0 mmol·L^− 1^ vs. placebo: 13.2 ± 2.5 mmol·L^− 1^; mean difference 1.2, 95% CI [− 0.14, 2.55], small effect, *d* = 0.43, 95% CI [− 0.044, 0.904]; *p* = 0.075). Similarly, mean heart rate throughout the Yo-Yo test tended to be higher in the caffeine trial although these differences were not statistically different (caffeine: 173 ± 7 b·min^− 1^ vs. placebo: 169 ± 14 b·min^− 1^; mean difference 3.6 b·min^− 1^, 95% CI [− 2.19, 9.48], small effect, *d* = 0.264, 95% CI [− 0.230, 0.750]; *p* = 0.204).

Individual responses on the battery of tests after caffeine supplementation are shown in Fig. 5.

## Discussion

The main findings of this study were that a low dose of caffeine supplied in gum enhanced performance on the CMJ by 3.6%, increased resistance to fatigue during a 6 × 30 m repeated sprint test, and improved performance on the Yo-Yo IR2 by 14.5%. However, caffeine gum failed to significantly improve performance in the Illinois agility test. Consistent with a number of previous studies there was evidence of variability in the response to caffeine among the participants. In 11 of 17 participants, caffeine supplementation improved CMJ, FI and the Yo-Yo IR2 test. For 5 participants, caffeine improved performance on two of the tests and for one participant, caffeine worsened performance in all the tests (see Fig. 5).

In the present study, caffeine evoked a small, statistically significant 3.6% increase in CMJ height. This magnitude of effect is consistent with previous studies using a similar dose of caffeine [4, 33] Clarke et al. [33] found that CMJ height during a rugby league simulation protocol was approximately 2% higher in elite rugby league players when they ingested 3 mg·kg^− 1^ of body weight of caffeine alongside 40 g of carbohydrate in comparison to carbohydrate alone. Del Coso et al. reported that 3 mg·kg^− 1^ of body weight of caffeine delivered in a commercial energy drink increased total muscle power output by 9% during a 15 s jumping test, in women from the Spanish rugby sevens national team. In contrast, in a couple of studies, caffeine has failed to exert a statistically significant effect on jumping ability [34, 35]. Tucker et al. [34] found no statistically significant effect of 3 mg·kg^− 1^ of caffeine on 10 vertical rebound jumps in male basketball players, although the sample size was very small (*n* = 5). Also, Stanjanovic et al. [35] found that in female basketball players, caffeine (3 mg·kg^− 1^) had no statistically significant effect on CMJ performed with and without an arm swing, although the effect sizes were slightly larger than the present study (0.29 and 0.30 versus 0.22).

There was no statistically significant effect of caffeine gum on performance in the 6 × 30 m sprint test. However, there was a statistically significant reduction in an index of fatigue indicating a more sustained capacity for repeated sprint performance. At the time of data collection there was only one study that had reported on the effect of caffeine gum on performance of a repeated sprint protocol. Russell et al. [24] investigated the effect of caffeine gum (400 mg of caffeine) in professional academy rugby players undertaking 2 x repeated sprint protocols (6 × 40 m) separated by a 15 min passive recovery period (to simulate half-time). Caffeine gum failed to attenuate the decrement in performance from the first set of sprints to the second. Direct comparison with the present results is difficult because Russell et al. [24] did not report a fatigue index and their protocol was designed to investigate the ability of caffeine to rescue the decrement in performance that accompanies passive recovery between bouts of high intensity exercise. After the current study was completed, Evans et al. [26] reported that 200 mg of caffeine delivered in gum did not significantly improve performance of team sport players (soccer, rugby and hockey) completing a 10 × 40 m sprint test. However, a secondary data analysis revealed that the gum exerted a significant beneficial effect in those participants with a low intake of caffeine (< 40 mg·d^− 1^), but not in those with a higher habitual intake (> 130 mg·d^− 1^). Interestingly, the group with a higher habitual intake of caffeine had a mean intake lower than the rugby players in the null study of Russell et al. [24]. Others have reported benefits of caffeine when it is administered using traditional modes of delivery [36, 37]. For example, Del Coso et al. [5] found a beneficial effect of a caffeinated energy drink (3 mg·kg^− 1^ of body weight) on mean peak running speed (+ 2.7%) in semi-professional soccer players completing 7 × 30 m sprints. Similarly, Carr and colleagues [37] reported that caffeine (6 mg·kg^− 1^ of body weight) improved short sprint performance (20 m with an 180^o^ turn at 10 m) in team sport players when recovery time between sprints was 5.5 s or 20 s. The findings of a more sustained capacity for repeated sprint performance observed in this trial agrees with these studies.

This is the first study to show that a low dose of caffeine (200 mg) supplied in gum can improve performance (+ 14%) on the Yo-Yo IR-2 test. Ranchordas et al. [4] previously reported a marginal 2% improvement in performance in soccer players undertaking a different version of the Yo-Yo test (IR-1) after the same dose of caffeinated gum. The magnitude of effect observed in the current study is very similar to Mohr and colleagues [38] who found a 16% increase in distance covered on the Yo-Yo IR-2 in team sport players administered a moderately high dose of caffeine (6 mg·kg^− 1^ of body weight) in capsule form.

Caffeine gum failed to significantly improve agility. In agreement with this null finding, Lorina et al. [39] reported that 6 mg·kg^− 1^ caffeine failed to enhance performance on The Pro Agility Run test in young adult males. Similarly, Astorina et al. [40] found that a low dose of caffeine (80 mg in a drink of Red Bull) and Lee et al. [41] found that a moderate dose of caffeine (6 mg·kg^− 1^) failed to improve performance of female team sports players in the repeated agility T test. In contrast, Duvnjak-Zaknich et al. [42] reported that in male team-sport athletes, 6 mg·kg^− 1^ of caffeine had potential benefits on reactive agility tests conducted before, during and after a simulated team sport fatiguing protocol. It should be noted that Duvnjak-Zaknich et al. [42] found limited evidence of a treatment effect using traditional statistical approaches, and clearer effects were only detected when their data was analysed using magnitude-based inferences (a contemporary statistical approach [43]).

It is likely that the improvements observed after caffeine gum in this study were mediated through antagonism of adenosine receptors [7]. Adenosine receptor antagonism may have improved jumping performance by enhancing motor unit recruitment and rate coding in lower body muscle groups as a result of excitatory effects in the supraspinal region [44]. Blocking the actions of adenosine also increases arousal and reduces the perception of pain [45] which may a have contributed to the greater distance covered in the Yo-Yo IR2 and resistance to fatigue during the sprint test.

The improvements observed in this study may transfer into match play. The increase in jump height, although small, may determine who wins possession during a line-out or when catching a ball after it has been kicked. A more sustained capacity for repeated sprint performance could allow rugby players to maintain sprint speeds towards the end of a match, when fatigue may impair performance. Rugby matches are characterised by high intensity bursts with short recovery times, so the increased total distance covered in the Yo-Yo IR2 test could translate into benefits during match play. An important consideration when using caffeine gum is that it should not be used during pre-, mid- and end-season testing to enhance performance on the testing battery, because this could influence the interpretation of the data for the coaching and conditioning staff.

### Limitations

This study has several limitations. Plasma caffeine was not measured in the participants however, plasma caffeine concentration has been shown to rise rapidly in response to caffeinated gum. Each piece of gum contained 100 mg of caffeine, so participants were provided with an absolute dose (200 mg) rather than a dose adjusted to bodyweight. This resulted in participants receiving a caffeine dose between 2.1 to 2.7 mg·kg^− 1^ of bodyweight. Moreover, there is individual variation in the extent of caffeine that is released from gum [46]. The range in relative dose and possible variable release of the caffeine from the gum may have contributed to the inter-individual variation in response, but more accurately models the ‘real world’ use of caffeine gum by athletes. It is possible that larger effects may have been observed if a higher dose of caffeine had been administered, however, the aim was to investigate a low dose to reduce the possibility of adverse effects and to follow the manufacturer’s recommendation for use. Low doses of caffeine have shown less consistent ergogenic effects than higher doses [47–49] . For example, Desbrow et al. [47] found no effect of 1.5 mg·kg^− 1^ and 3 mg·kg^− 1^ of caffeine on cycle time trial performance. Jenkins et al. [48] reported that 2 and 3 mg·kg^− 1^ of caffeine improved cycling performance, but 1 mg·kg^− 1^ had no effect. Another limitation of this study was that all four performance tests were conducted on the same day with 3–5 min recovery periods in between each test. It is unlikely that the CMJ test and the Illinois agility test performed at the start would have caused fatigue and negatively affected the repeated sprint and Yo-Yo-IR2 tests. However, the repeated sprint test may have affected performance on the subsequent Yo-Yo test. Nevertheless, the aim was to investigate the effects of caffeine on endurance performance in a fatigued state to simulate the demands of competition.

## Conclusions

This study demonstrated that a low dose of caffeine (200 mg) supplied in gum enhances performance of university standard male rugby players undertaking a battery of rugby specific tests. Moreover, the onset of action was rapid, occurring in performances tests that commenced 5 min after the gum was chewed. This rapid onset of action aligns with the pharmacokinetic study of Kammiori et al. and very recent work in the authors’ laboratory [46]. Such a rapid onset of action has practical application to rugby (and other team sports), because there is often limited time for nutrition intervention during matches such as at half-time and before substitutes enter play. The finding that a low dose of caffeine enhanced performance is practically important, because low doses are less likely to produce adverse effects, which may limit the use of higher doses. Caffeine gum may be less likely to cause gastric irritation than capsules, because a substantial proportion of the dose is absorbed across the buccal mucosa. Accordingly, gum may be useful for players who cannot tolerate caffeinated beverages or capsules before kick-off, because of gastrointestinal distress.

## References

[1] Del Coso J, Muñoz G, Muñoz-Guerra J (2011). Prevalence of caffeine use in elite athletes following its removal from the World Anti-Doping Agency list of banned substances. Appl Physiol Nutr Metab.

[2] Ganio MS, Klau JF, Casa DJ, Armstrong LE, Maresh CM (2009). Effect of caffeine on sport-specific endurance performance: a systematic review. J Strength Cond Res.

[3] Astorino TA, Roberson DW (2010). Efficacy of acute caffeine ingestion for short-term high-intensity exercise performance: a systematic review. J Strength Cond Res.

[4] Ranchordas MK, King G, Russell M, Lynn A, Russell M. Effects of caffeinated gum on a battery of soccer-specific tests in trained university-standard male soccer players. Int J Sport Nutr Exerc Metab. 2018:1–18. 10.1123/ijsnem.2017-0405 29584462

[5] Del Coso J, Munoz-Fernandez VE, Munoz G, Fernandez-Elias VE, Ortega JF, Hamouti N, Barbero JC, Munoz-Guerra J (2012). Effects of a caffeine-containing energy drink on simulated soccer performance. PLoS One.

[6] Burke LM (2008). Caffeine and sports performance. Appl Physiol Nutr Metab.

[7] Kalmar JM, Cafarelli E (2004). Caffeine: a valuable tool to study central fatigue in humans?. Exerc Sport Sci Rev.

[8] Davis JK, Green JM (2009). Caffeine and anaerobic performance: ergogenic value and mechanisms of action. Sports Med.

[9] Santos VG, Santos VR, Felippe LJ, Almeida JW, Bertuzzi R, Kiss MA, Lima-Silva AE (2014). Caffeine reduces reaction time and improves performance in simulated-contest of taekwondo. Nutrients.

[10] Jordan JB, Korgaokar A, Farley RS, Coons JM, Caputo JL (2014). Caffeine supplementation and reactive agility in elite youth soccer players. Pediatr Exerc Sci.

[11] Roberts SP, Trewartha G, Higgitt RJ, El-Abd J, Stokes KA (2008). The physical demands of elite English rugby union. J Sports Sci.

[12] Quarrie KL, Cantu RC, Chalmers DJ (2002). Rugby union injuries to the cervical spine and spinal cord. Sports Med.

[13] Austin D, Gabbett T, Jenkins D (2011). Repeated high-intensity exercise in professional rugby union. J Sports Sci.

[14] Coughlan GF, Green BS, Pook PT, Toolan E, O'Connor SP (2011). Physical game demands in elite rugby union: a global positioning system analysis and possible implications for rehabilitation. J Orthop Sports Phys Ther.

[15] Cunniffe B, Proctor W, Baker JS, Davies B (2009). An evaluation of the physiological demands of elite rugby union using global positioning system tracking software. J Strength Cond Res.

[16] Del Coso J, Portillo J, Muñoz G, Abián-Vicén J, Gonzalez-Millán C, Muñoz-Guerra J (2013). Caffeine-containing energy drink improves sprint performance during an international rugby sevens competition. Amino Acids.

[17] Stuart GR, Hopkins WG, Cook C, Cairns SP (2005). Multiple effects of caffeine on simulated high-intensity team-sport performance. Med Sci Sports Exerc.

[18] Magkos F, Kavouras SA (2005). Caffeine use in sports, pharmacokinetics in man, and cellular mechanisms of action. Crit Rev Food Sci Nutr.

[19] Graham T, Hibbert E, Sathasivam P (1998). Metabolic and exercise endurance effects of coffee and caffeine ingestion. J Appl Physiol.

[20] Bloms LP, Fitzgerald JS, Short MW, Whitehead JR (2016). The effects of caffeine on vertical jump height and execution in collegiate athletes. J Strength Cond Res.

[21] Kamimori GH, Karyekar CS, Otterstetter R, Cox DS, Balkin TJ, Belenky GL, Eddington ND (2002). The rate of absorption and relative bioavailability of caffeine administered in chewing gum versus capsules to normal healthy volunteers. Int J Pharm.

[22] Paton CD, Lowe T, Irvine A (2010). Caffeinated chewing gum increases repeated sprint performance and augments increases in testosterone in competitive cyclists. Eur J Appl Physiol.

[23] Chen M, Shah VP, Crommelin DJ, Shargel L, Bashaw D, Bhatti M, Blume H, Dressman J, Ducharme M, Fackler P (2011). Harmonization of regulatory approaches for evaluating therapeutic equivalence and interchangeability of multisource drug products: workshop summary report. Eur J Pharm Sci.

[24] Russell M, Reynolds N, Crewther B, Cook C, Kilduff L. The physiological and performance effects of caffeine gum consumed during a simulated half-time by professional academy Rugby union players. J Strength Cond Res. 2018. 10.1519/JSC.0000000000002185 29210957

[25] Bell DG, McLellan TM (2002). Exercise endurance 1, 3, and 6 h after caffeine ingestion in caffeine users and nonusers. J Appl Physiol (1985).

[26] Evans M, Tierney P, Gray N, Hawe G, Macken M, Egan B (2018). Acute ingestion of caffeinated chewing gum improves repeated Sprint performance of team sport athletes with low habitual caffeine consumption. Int J Sport Nutr Exerc Metab.

[27] Markovic G, Dizdar D, Jukic I, Cardinale M (2004). Reliability and factorial validity of squat and countermovement jump tests. The Journal of Strength & Conditioning Research.

[28] Hachana Y, Chaabene H, Nabli MA, Attia A, Moualhi J, Farhat N, Elloumi M (2013). Test-retest reliability, criterion-related validity, and minimal detectable change of the Illinois agility test in male team sport athletes. J Strength Cond Res.

[29] Spencer M, Fitzsimons M, Dawson B, Bishop D, Goodman C (2006). Reliability of a repeated-sprint test for field-hockey. J Sci Med Sport.

[30] Bangsbo J, Iaia FM, Krustrup P (2008). The Yo-Yo intermittent recovery test. Sports Med.

[31] Borg GA (1982). Psychophysical bases of perceived exertion. Med Sci Sports Exerc.

[32] Cohen J (1988). Statistical power analysis for the behavioral sciences.

[33] Clarke JS, Highton J, Close GL, Twist C. Carbohydrate and caffeine improves high intensity running of elite rugby league interchange players during simulated match play. J Strength Cond Res. 2016. 10.1519/JSC.0000000000001742 27930447

[34] Tucker MA, Hargreaves JM, Clarke JC, Dale DL, Blackwell GJ (2013). The effect of caffeine on maximal oxygen uptake and vertical jump performance in male basketball players. The Journal of Strength & Conditioning Research.

[35] Stojanovic E, Stojiljkovic N, Scanlan AT, Dalbo VJ, Stankovic R, Antic V, Milanovic Z: Acute caffeine supplementation promotes small to moderate improvements in performance tests indicative of in-game success in professional female basketball players. Appl Physiol Nutr Metab 2019, (ja). 10.1139/apnm-2018-0671 30633542

[36] Del Coso J, Salinero JJ, González-Millán C, Abián-Vicén J, Pérez-González B (2012). Dose response effects of a caffeine-containing energy drink on muscle performance: a repeated measures design. Journal of the International Society of Sports Nutrition.

[37] Carr A, Dawson B, Schneiker K, Goodman C, Lay B (2008). Effect of caffeine supplementation on repeated sprint running performance. J Sports Med Phys Fitness.

[38] Mohr M, Nielsen JJ, Bangsbo J (2011). Caffeine intake improves intense intermittent exercise performance and reduces muscle interstitial potassium accumulation. J Appl Physiol (1985).

[39] Lorino AJ, Lloyd LK, Crixell SH, Walker JL (2006). The effects of caffeine on athletic agility. J Strength Cond Res.

[40] Astorino TA, Matera AJ, Basinger J, Evans M, Schurman T, Marquez R (2012). Effects of red bull energy drink on repeated sprint performance in women athletes. Amino Acids.

[41] Lee CL, Cheng CF, Astorino TA, Lee CJ, Huang HW, Chang WD (2014). Effects of carbohydrate combined with caffeine on repeated sprint cycling and agility performance in female athletes. J Int Soc Sports Nutr.

[42] Duvnjak-Zaknich DM, Dawson BT, Wallman KE, Henry G (2011). Effect of caffeine on reactive agility time when fresh and fatigued. Med Sci Sports Exerc.

[43] Hopkins WG, Marshall SW, Batterham AM, Hanin J (2009). Progressive statistics for studies in sports medicine and exercise science. Med Sci Sports Exerc.

[44] Behrens M, Mau-Moeller A, Weippert M, Fuhrmann J, Wegner K, Skripitz R, Bader R, Bruhn S (2015). Caffeine-induced increase in voluntary activation and strength of the quadriceps muscle during isometric, concentric and eccentric contractions. Sci Rep.

[45] Davis JM, Zhao Z, Stock HS, Mehl KA, Buggy J, Hand GA (2003). Central nervous system effects of caffeine and adenosine on fatigue. Am J Physiol Regul Integr Comp Physiol.

[46] Morris C, Viriot SM, Mirza QUF, Morris G, Lynn A. Caffeine release and absorption from caffeinated gums. Food Funct. 2019. 10.1039/c9fo00431a 30919868

[47] Desbrow B, Barrett CM, Minahan CL, Grant GD, Leveritt MD (2009). Caffeine, cycling performance, and exogenous CHO oxidation: a dose-response study. Med Sci Sports Exerc.

[48] Jenkins NT, Trilk JL, Singhal A, O’Connor PJ, Cureton KJ (2008). Ergogenic effects of low doses of caffeine on cycling performance. Int J Sport Nutr Exerc Metab.

[49] Ryan EJ, Kim C, Muller MD, Bellar DM, Barkley JE, Bliss MV, Jankowski-Wilkinson A, Russell M, Otterstetter R, Macander D (2012). Low-dose caffeine administered in chewing gum does not enhance cycling to exhaustion. J Strength Cond Res.

